# The hypoxic tumor microenvironment and drug resistance against EGFR inhibitors: preclinical study in cetuximab-sensitive head and neck squamous cell carcinoma cell lines

**DOI:** 10.1186/s13104-015-1197-6

**Published:** 2015-06-02

**Authors:** Carolien Boeckx, Jolien Van den Bossche, Ines De Pauw, Marc Peeters, Filip Lardon, Marc Baay, An Wouters

**Affiliations:** Center for Oncological Research (CORE) Antwerp, University of Antwerp, Universiteitsplein 1, 2610 Wilrijk, Belgium; Department of Oncology, Antwerp University Hospital, Wilrijkstraat 10, 2650 Edegem, Belgium

**Keywords:** Cetuximab, Erlotinib, Hypoxia, Cytotoxicity

## Abstract

**Background:**

Increased expression of the epidermal growth factor receptor (EGFR) is observed in more than 90% of all head and neck squamous cell carcinomas (HNSCC). Therefore, EGFR has emerged as a promising therapeutic target. Nevertheless, drug resistance remains a major challenge and an important potential mechanism of drug resistance involves the hypoxic tumor microenvironment. Therefore, we investigated the cytotoxic effect of the EGFR-targeting agents cetuximab and erlotinib under normoxia versus hypoxia.

**Findings:**

Three cetuximab-sensitive HNSCC cell lines (SC263, LICR-HN2 and LICR-HN5) were treated with either cetuximab or erlotinib. Cells were incubated under normal or reduced oxygen conditions (<0.1% O_2_) for 24 or 72 h immediately after drug addition. Cell survival was assessed with the sulforhodamine B assay. Cetuximab and erlotinib established a dose-dependent growth inhibition under both normal and prolonged reduced oxygen conditions in all three HNSCC cell lines. However, a significantly increased sensitivity to cetuximab was observed in SC263 cells exposed to hypoxia for 72 h (p = 0.05), with IC_50_ values of 2.38 ± 0.59 nM, 0.64 ± 0.38 nM, and 0.10 ± 0.05 nM under normoxia, hypoxia for 24 h and hypoxia for 72 h, respectively. LICR-HN5 cells showed an increased sensitivity towards erlotinib when cells were incubated under hypoxia for 24 h (p = 0.05).

**Conclusions:**

Our results suggest that both EGFR-inhibitors cetuximab and erlotinib maintain their growth inhibitory effect under hypoxia. These results suggest that resistance to anti-EGFR therapy in HNSCC is probably not the result of hypoxic regions within the tumor and other mechanisms are involved.

## Background

Personalized medicine using targeted therapies is a promising alternative to the current conventional treatment strategies (surgery, chemotherapy and radiation), which are only effective in 50% of head and neck squamous cell carcinoma (HNSCC) patients [1]. Increased expression of the epidermal growth factor receptor (EGFR) is frequently observed in most HNSCC and leads to a number of cellular processes involved in proliferation, differentiation, anti-apoptotic signaling, angiogenesis and metastasis, thereby driving the malignant behavior of tumor cells [2–4]. Consequently, therapies targeting EGFR are among the most promising molecular therapeutics for HNSCC. However, drug resistance limits the clinical efficacy of these EGFR targeting agents and no predictive biomarker has entered the clinic yet. Although the response rate to the anti-EGFR directed monoclonal antibody cetuximab as a monotherapy is as low as 10–13%, it does provide a clinical benefit when used in conjunction with radiation alone or in combination with chemotherapy [5–7]. The lack of clinical responses to EGFR-directed therapies may be caused by multiple intrinsic and extrinsic/acquired resistance mechanisms that can compensate for reduced EGFR signaling and/or modulate EGFR-dependent signaling [8]. In HNSCC, however, no consistent genetic alteration appears to confer resistance or sensitivity to these EGFR targeting agents [8]. One of the important potential mechanisms of drug resistance is attributable to the tumor microenvironment and many components of this microenvironment are potential targets for therapeutic interventions.

Tumor hypoxia, or low oxygen concentration, is a result of disordered vasculature found in almost all solid tumors, including HNSCC, and is evoked by rapid rate of tumor growth, poor tumor perfusion or transiently disrupted tumor blood flow. Cells exposed to prolonged hypoxia accumulate changes in their growth properties and DNA, leading to chemo- and radioresistance and enhanced metastatic potential [9, 10]. In HNSCC patients, tumor hypoxia has been identified as a negative prognostic factor [11, 12].

Tumor cells will adapt quickly to the hypoxic stress by regulating the expression of various genes such as hypoxia-inducible factors (HIFs) and vascular endothelial growth factor (VEGF). The HIFs are transcription factors that play an essential role in the cellular response to hypoxic stress, as they are able to activate the EGFR signaling pathway. Several studies have shown that hypoxic regions within the tumor have an increased expression of EGFR compared to normoxic regions [13–15]. Hypoxia activated EGFR signaling in turn stimulates HIF signaling to improve cellular survival, induces epithelial to mesenchymal transition (EMT) and activates AKT, a downstream EGFR signaling molecule [16–19]. All these factors maintain or contribute to the malignant behavior of the tumor. An in-depth summary describing the crosstalk between EGFR and hypoxia is beyond the scope of this introduction. For a more detailed description, we recommend the extensive review by Wouters et al. [20].

Given the link between hypoxia and EGFR signaling, we hypothesized that hypoxia and its subsequent signaling might be responsible for anti-EGFR drug resistance. Therefore, the present study investigates cell survival after treatment with the EGFR-targeting monoclonal antibody cetuximab and the EGFR tyrosine kinase inhibitor erlotinib under normoxic versus hypoxic conditions, in three cetuximab-sensitive HNSCC cell lines.

## Methods

### Cell culture

Three cetuximab-sensitive, EGFRvIII negative HNSCC cell lines SC263, LICR-HN2 and LICR-HN5 were included in this study. All cell lines were grown as monolayers in Dulbecco’s Modified Eagle Medium (DMEM), supplemented with 10% fetal calf serum, 2 mM glutamine and 1% penicillin/streptomycin. All media and supplements were obtained from Invitrogen (Merelbeke, Belgium). Cultures were maintained in exponential growth in a humidified 5% CO_2_/95% air atmosphere at 37°C. Cells were tested for mycoplasma infection through regular testing (MycoAlert™, Plus Mycoplasma detection kit, Lonza, Verviers, Belgium).

### Oxygen conditions

Hypoxic conditions (0% O_2_, 5% CO_2_, 95% N_2_) were achieved in a Bactron IV anaerobic chamber (Shel Lab, Cornelius, OR, USA) and we used the hypoxia model previously optimized and characterized [21, 22]. Measurements with ToxiRae II air oxymeter (Rae BeNeLux, Hoogstraten, Belgium) confirmed that the oxygen tension in the gas phase was stable at <0.1% O_2_. Hypoxic incubation was initiated after cells had been cultured under normoxic conditions overnight, allowing attachment to culture dishes.

### Pharmaceuticals

Cetuximab (Merck, Darmstadt, Germany) was diluted in sterile PBS and erlotinib (Selleck Chemicals, Houston, USA) was diluted in DMSO.

### Growth inhibition experiments

Cytotoxicity studies were performed in 48 well plates and optimal seeding densities for each cell line were determined to ensure exponential growth during the assay. After an overnight recovery period, cells were treated either with 0–10 nM cetuximab for 168 h or with 0–20 μM erlotinib for 72 h. Cells were incubated under normal or reduced oxygen conditions for 24 or 72 h immediately after addition of the drug. After incubation, cell survival was evaluated by the sulphorhodamine B assay, as previously described [23]. Optical density (OD) was determined at 540 nM with the iMark microplate reader (Bio-Rad, Temse, Belgium). The percentage of cell survival was calculated for each concentration by the following formula: (OD_treated_/OD_untreated_) × 100. The IC_50_ value, representing the drug concentration reducing cell growth to 50%, was calculated using WinNonlin software with pharmacodynamic model 107. All experiments were performed in triplicate.

### Statistical analysis

All experiments were performed at least in triplicate. Results are presented as mean ± standard deviation. Possible significant differences (p ≤ 0.05) were evaluated with Mann–Whitney U test using SPSS v20.0 software (Chicago, IL, USA).

## Results

As the presence of tumor hypoxia may confer resistance to EGFR inhibitors, we performed growth inhibition experiments under normoxic and hypoxic conditions for the tyrosine kinase inhibitor erlotinib and the monoclonal antibody cetuximab on three human, cetuximab sensitive HNSCC cell lines.

### Cytotoxicity of the tyrosine kinase inhibitor erlotinib, under normoxic and hypoxic conditions

**Figure 1:**
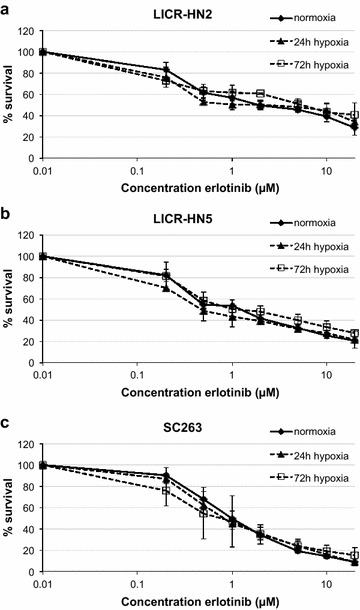
Cytotoxic effect of erlotinib. Dose–response curves of LICR-HN2 (**a**), LICR-HN5 (**b**) and SC263 (**c**) cells after 72 h of erlotinib treatment under normoxia and hypoxia (24 and 72 h).

The cytotoxicity profiles of the HNSCC cell lines under normoxic and hypoxic conditions (24 and 72 h) for erlotinib (0–20 µM) are shown in Figure 1. IC_50_ values of these three oxygen conditions were calculated for each cell line (Table 1).

**Table 1:** IC_50_ values (μM) of erlotinib treatment in three HNSCC cell lines (mean ± standard deviation)

Cell line	Normoxia	24 h hypoxia	72 h hypoxia
LICR-HN2	2.26 ± 0.83	1.06 ± 1.42	3.34 ± 1.25
LICR-HN5	1.16 ± 0.35	0.60 ± 0.23	1.49 ± 0.60
SC263	1.05 ± 0.12	0.95 ± 0.16	0.81 ± 0.12

Erlotinib established a dose-dependent growth inhibition under both normal and reduced oxygen conditions in all three HNSCC cell lines. Furthermore, LICR-HN5 cells were more sensitive to erlotinib when exposed to hypoxia for 24 h compared with normoxic LICR-HN5 cells (p = 0.05). However, this increased sensitivity to erlotinib was not observed when exposed to hypoxia for 72 h. As such, our results suggest that resistance to erlotinib could not be elicited by hypoxic incubation for 24 or 72 h.

### Cytotoxicity of the monoclonal antibody cetuximab, under normoxic and hypoxic conditions

**Figure 2:**
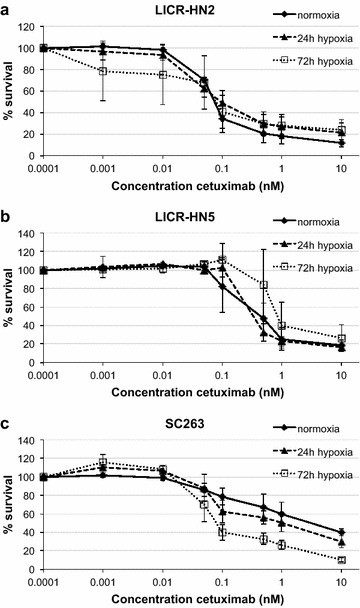
Cytotoxic effect of cetuximab. Dose–response curves of LICR-HN2 (**a**), LICR-HN5 (**b**) and SC263 (**c**) cells after 168 h of cetuximab treatment under normoxia and hypoxia (24 and 72 h).

The cytotoxicity profiles of the HNSCC cell lines under normoxic and hypoxic conditions (24 and 72 h) for cetuximab (0–10 nM) are shown in Figure 2. IC_50_ values of these three oxygen conditions were calculated for each cell line (Table 2).

**Table 2:** IC_50_ values (nM) of cetuximab treatment in three HNSCC cell lines (mean ± standard deviation)

Cell line	Normoxia	24 h hypoxia	72 h hypoxia
LICR-HN2	0.08 ± 0.03	0.12 ± 0.05	0.08 ± 0.08
LICR-HN5	0.42 ± 0.10	0.37 ± 0.09	1.17 ± 0.47
SC263	2.38 ± 0.59	0.64 ± 0.38	0.10 ± 0.05

The effect of cetuximab on cell survival was not affected by the presence or absence of oxygen in LICR-HN2 and LICR-HN5 cells. However, SC263 cells were more sensitive to higher concentrations of cetuximab when cells were incubated under reduced oxygen tension for 24 or 72 h, resulting in significantly decreased IC_50_ values (p = 0.05 in both cases). Overall, our results showed that no therapeutic resistance towards cetuximab could be evoked by prolonged hypoxia in these HNSCC cells.

## Discussion

As the tumor and its surrounding microenvironment can affect each other and the (extremely) low prevalence of *EGFR* and *K*-*Ras* mutations in HNSCC would likely preclude a major role for these mutations as predictive biomarker [24, 25], drug resistance might occur from the tumor microenvironment. Furthermore, this microenvironment is often hypoxic. Therefore, we hypothesized that hypoxia might induce anti-EGFR therapeutic resistance. To test this hypothesis, we evaluated the cytotoxicity of the EGFR-blocking monoclonal antibody cetuximab and the small molecule EGFR tyrosine kinase inhibitor erlotinib in three HNSCC cell lines under hypoxic conditions for 24 and 72 h. We previously validated induction of HIF-1α and its downstream targets as well as induction of HIF activity in our experimental model [21].

In HNSCC patients, high levels of hypoxia-associated factors are associated with relapse following induction therapy that included cetuximab, and co-localization of EGFR and hypoxia markers are associated with poor outcome [12, 26]. With regard to resistance towards EGFR therapy, HIF-1α, the regulatory subunit of the HIF-1 transcription factor, is an important protein, as increased expression of HIF-1α has been reported to confer resistance to cetuximab in human vulvar squamous carcinoma cells and downregulation of HIF-1 alpha is required for cetuximab-induced anti-proliferative effects [27, 28].

In contrast, however, our study demonstrated that prolonged hypoxia (24 and 72 h) did not induce resistance towards cetuximab and erlotinib therapy in three HNSCC cell lines. Therefore, no predictive biomarkers with regard to drug resistance and hypoxia could be identified. In line with our observations, only few papers were able to illustrate hypoxia-induced treatment resistance [26, 29] and most studies on EGFR-targeting agents supported a markedly increased antitumor potency of both monoclonal antibodies and tyrosine kinase inhibitors under hypoxic conditions [20, 30, 31].

Concerning the EGFR-targeted monoclonal antibodies, it has been speculated that hypoxia enhances the sensitivity to the cytotoxic effect of these drugs. For example, cetuximab was more cytotoxic against hypoxic than well-oxygenated A431 lung cancer cells grown in vitro and it reduced the overexpression of hypoxia markers like HIF-1α, CA9 and VEGF [32]. In addition, it was observed that cetuximab could clearly downregulate HIF-1α levels in cancer cells that were sensitive to EGFR inhibition and it was shown that HIF-1α was required, although it might not be sufficient, to mediate the response of cancer cells to cetuximab [27, 28, 33]. Furthermore, radiosensitization of HNSCC cell lines is shown to be partly attributable to inhibition of radiation-induced upregulation of HIF-1α [34]. Moreover, together with the demonstrated antiproliferative and proapoptotic effects, the antiangiogenic activity of cetuximab is now believed to contribute to its overall antitumor activity in vivo. For example, immunohistochemical analysis of HNSCC tumor xenografts after systemic administration of cetuximab demonstrated inhibition of the expression of tumor angiogenesis markers, including VEGF and Factor VIII [35].

Similarly, considering the effect of EGFR-targeting tyrosine kinase inhibitors under reduced oxygen conditions, several studies indicated that treatment with gefitinib or erlotinib was associated with a dramatic reduction in the proportion of viable hypoxic tumor cells [27, 28, 31, 36–40]. These effects are, at least in part, attributable to decreased VEGF production and secretion, decreased production of HIF-1α and increased vascular normalization [27, 28, 31, 36–40]. For example, Pore et al. [38] demonstrated that gefitinib and erlotinib decreased VEGF mRNA expression and decreased the secretion of VEGF protein in response to hypoxia in SQ20B HNSCC cells. Likewise, treatment of human HNSCC xenografts with gefitinib significantly reduced vessel formation and inhibited the early angiogenic process by targeting endothelial cells [41], ultimately resulting in vascular normalization, improved blood flow and thus improved oxygenation.

## Conclusions

Our results suggest that both anti-EGFR therapeutics cetuximab and erlotinib maintain their efficacy both under 24 h and 72 h of reduced oxygen tension in the HNSCC cell lines included in our study. Furthermore, an increased sensitivity to cetuximab was observed in SC263 cells exposed to hypoxia for 72 h and LICR-HN5 cells exhibited an increased sensitivity to erlotinib when exposed to hypoxia for 24 h. Our results, therefore, suggest that resistance to anti-EGFR therapy in HNSCC is most likely not the result of hypoxic regions within the tumor and, hence, other mechanisms are involved. One important limitation of our in vitro study is the lack of the real microenvironment that surrounds tumors in vivo. As our research was based on cell lines in vitro, no other factors of the tumor microenvironment were taken into account. Therefore, further studies using tumor animal models would certainly be warranted.

## References

[1] Marur S, Forastiere AA (2008). Head and neck cancer: changing epidemiology, diagnosis, and treatment. Mayo Clin Proc.

[2] Rubin Grandis J, Melhem MF, Gooding WE, Day R, Holst VA, Wagener MM (1998). Levels of TGF-alpha and EGFR protein in head and neck squamous cell carcinoma and patient survival. J Nat Cancer Inst.

[3] Grandis JR, Sok JC (2004). Signaling through the epidermal growth factor receptor during the development of malignancy. Pharmacol Ther.

[4] Mrhalova M, Plzak J, Betka J, Kodet R (2005). Epidermal growth factor receptor—its expression and copy numbers of EGFR gene in patients with head and neck squamous cell carcinomas. Neoplasma.

[5] Cunningham D, Humblet Y, Siena S, Khayat D, Bleiberg H, Santoro A (2004). Cetuximab monotherapy and cetuximab plus irinotecan in irinotecan-refractory metastatic colorectal cancer. N Engl J Med.

[6] Bonner JA, Harari PM, Giralt J, Azarnia N, Shin DM, Cohen RB (2006). Radiotherapy plus cetuximab for squamous-cell carcinoma of the head and neck. N Engl J Med.

[7] Vermorken JB, Trigo J, Hitt R, Koralewski P, Diaz-Rubio E, Rolland F (2007). Open-label, uncontrolled, multicenter phase II study to evaluate the efficacy and toxicity of cetuximab as a single agent in patients with recurrent and/or metastatic squamous cell carcinoma of the head and neck who failed to respond to platinum-based therapy. J Clin Oncol.

[8] Boeckx C, Baay M, Wouters A, Specenier P, Vermorken JB, Peeters M (2013). Anti-epidermal growth factor receptor therapy in head and neck squamous cell carcinoma: focus on potential molecular mechanisms of drug resistance. Oncologist.

[9] Brizel DM, Dodge RK, Clough RW, Dewhirst MW (1999). Oxygenation of head and neck cancer: changes during radiotherapy and impact on treatment outcome. Radiother Oncol.

[10] Gabalski EC, Adam M, Pinto H, Brown JM, Bloch DA, Terris DJ (1998). Pretreatment and midtreatment measurement of oxygen tension levels in head and neck cancers. Laryngoscope.

[11] Dunst J, Stadler P, Becker A, Lautenschlager C, Pelz T, Hansgen G (2003). Tumor volume and tumor hypoxia in head and neck cancers. The amount of the hypoxic volume is important. Strahlenther Onkol.

[12] Hoogsteen IJ, Marres HA, van den Hoogen FJ, Rijken PF, Lok J, Bussink J (2012). Expression of EGFR under tumor hypoxia: identification of a subpopulation of tumor cells responsible for aggressiveness and treatment resistance. Int J Radiat Oncol Biol Phys.

[13] Laderoute KR, Grant TD, Murphy BJ, Sutherland RM (1992). Enhanced epidermal growth factor receptor synthesis in human squamous carcinoma cells exposed to low levels of oxygen. Int J Cancer.

[14] Krause M, Ostermann G, Petersen C, Yaromina A, Hessel F, Harstrick A (2005). Decreased repopulation as well as increased reoxygenation contribute to the improvement in local control after targeting of the EGFR by C225 during fractionated irradiation. Radiother Oncol.

[15] Swinson DE, O’Byrne KJ (2006). Interactions between hypoxia and epidermal growth factor receptor in non-small-cell lung cancer. Clin Lung Cancer.

[16] Semenza GL, Agani F, Feldser D, Iyer N, Kotch L, Laughner E (2000). Hypoxia, HIF-1, and the pathophysiology of common human diseases. Adv Exp Med Biol.

[17] Franovic A, Gunaratnam L, Smith K, Robert I, Patten D, Lee S (2007). Translational up-regulation of the EGFR by tumor hypoxia provides a nonmutational explanation for its overexpression in human cancer. Proc Natl Acad Sci USA.

[18] Misra A, Pandey C, Sze SK, Thanabalu T (2012). Hypoxia activated EGFR signaling induces epithelial to mesenchymal transition (EMT). PLoS One.

[19] Stegeman H, Kaanders JH, Wheeler DL, van der Kogel AJ, Verheijen MM, Waaijer SJ (2012). Activation of AKT by hypoxia: a potential target for hypoxic tumors of the head and neck. BMC Cancer.

[20] Wouters A, Boeckx C, Vermorken JB, Van den Weyngaert D, Peeters M, Lardon F (2013). The intriguing interplay between therapies targeting the epidermal growth factor receptor, the hypoxic microenvironment and hypoxia-inducible factors. Curr Pharm Des.

[21] Wouters A, Pauwels B, Burrows N, Baay M, Deschoolmeester V, Vu TN (2014). The radiosensitising effect of gemcitabine and its main metabolite dFdU under low oxygen conditions is in vitro not dependent on functional HIF-1 protein. BMC Cancer.

[22] Wouters A, Pauwels B, Lambrechts HA, Pattyn GG, Ides J, Baay M (2009). Chemoradiation interactions under reduced oxygen conditions: cellular characteristics of an in vitro model. Cancer Lett.

[23] Pauwels B, Korst AE, de Pooter CM, Pattyn GG, Lambrechts HA, Baay MF (2003). Comparison of the sulforhodamine B assay and the clonogenic assay for in vitro chemoradiation studies. Cancer Chemother Pharmacol.

[24] Van Damme N, Deron P, Van Roy N, Demetter P, Bols A, Van Dorpe J (2010). Epidermal growth factor receptor and K-RAS status in two cohorts of squamous cell carcinomas. BMC Cancer.

[25] Szabo B, Nelhubel GA, Karpati A, Kenessey I, Jori B, Szekely C (2011). Clinical significance of genetic alterations and expression of epidermal growth factor receptor (EGFR) in head and neck squamous cell carcinomas. Oral Oncol.

[26] Byers LA, Holsinger FC, Kies MS, William WN, El-Naggar AK, Lee JJ (2010). Serum signature of hypoxia-regulated factors is associated with progression after induction therapy in head and neck squamous cell cancer. Mol Cancer Ther.

[27] Luwor RB, Lu Y, Li X, Mendelsohn J, Fan Z (2005). The antiepidermal growth factor receptor monoclonal antibody cetuximab/C225 reduces hypoxia-inducible factor-1 alpha, leading to transcriptional inhibition of vascular endothelial growth factor expression. Oncogene.

[28] Li X, Lu Y, Liang K, Pan T, Mendelsohn J, Fan Z (2008). Requirement of hypoxia-inducible factor-1alpha down-regulation in mediating the antitumor activity of the anti-epidermal growth factor receptor monoclonal antibody cetuximab. Mol Cancer Ther.

[29] Lee CM, Tannock IF (2010). The distribution of the therapeutic monoclonal antibodies cetuximab and trastuzumab within solid tumors. BMC Cancer.

[30] Riesterer O, Mason KA, Raju U, Yang Q, Wang L, Hittelman WN (2009). Enhanced response to C225 of A431 tumor xenografts growing in irradiated tumor bed. Radiother Oncol.

[31] Cerniglia GJ, Pore N, Tsai JH, Schultz S, Mick R, Choe R (2009). Epidermal growth factor receptor inhibition modulates the microenvironment by vascular normalization to improve chemotherapy and radiotherapy efficacy. PLoS One.

[32] Riesterer O, Yang Q, Raju U, Torres M, Molkentine D, Patel N (2011). Combination of anti-IGF-1R antibody A12 and ionizing radiation in upper respiratory tract cancers. Int J Radiat Oncol Biol Phys.

[33] Li X, Fan Z (2010). The epidermal growth factor receptor antibody cetuximab induces autophagy in cancer cells by downregulating HIF-1alpha and Bcl-2 and activating the beclin 1/hVps34 complex. Cancer Res.

[34] Lu H, Liang K, Lu Y, Fan Z (2012). The anti-EGFR antibody cetuximab sensitizes human head and neck squamous cell carcinoma cells to radiation in part through inhibiting radiation-induced upregulation of HIF-1alpha. Cancer Lett.

[35] Huang SM, Harari PM (2000). Modulation of radiation response after epidermal growth factor receptor blockade in squamous cell carcinomas: inhibition of damage repair, cell cycle kinetics, and tumor angiogenesis. Clin Cancer Res.

[36] Perrotte P, Matsumoto T, Inoue K, Kuniyasu H, Eve BY, Hicklin DJ (1999). Anti-epidermal growth factor receptor antibody C225 inhibits angiogenesis in human transitional cell carcinoma growing orthotopically in nude mice. Clin Cancer Res.

[37] Hirata A, Ogawa S, Kometani T, Kuwano T, Naito S, Kuwano M (2002). ZD1839 (Iressa) induces antiangiogenic effects through inhibition of epidermal growth factor receptor tyrosine kinase. Cancer Res.

[38] Pore N, Jiang Z, Gupta A, Cerniglia G, Kao GD, Maity A (2006). EGFR tyrosine kinase inhibitors decrease VEGF expression by both hypoxia-inducible factor (HIF)-1-independent and HIF-1-dependent mechanisms. Cancer Res.

[39] Rho JK, Choi YJ, Lee JK, Ryoo BY, Na II, Yang SH (2009). Gefitinib circumvents hypoxia-induced drug resistance by the modulation of HIF-1alpha. Oncology Rep.

[40] Luo HY, Wei W, Shi YX, Chen XQ, Li YH, Wang F (2010). Cetuximab enhances the effect of oxaliplatin on hypoxic gastric cancer cell lines. Oncol Rep.

[41] Huang SM, Li J, Armstrong EA, Harari PM (2002). Modulation of radiation response and tumor-induced angiogenesis after epidermal growth factor receptor inhibition by ZD1839 (Iressa). Cancer Res.

